# Design of V-shaped cantilevers for enhanced multifrequency AFM measurements

**DOI:** 10.3762/bjnano.11.135

**Published:** 2020-10-06

**Authors:** Mehrnoosh Damircheli, Babak Eslami

**Affiliations:** 1 Department of Mechanical Engineering, Widener University, Chester, Pennsylvania, 19013, USA; 2 Department of Mechanical Engineering, Shahr-e-Qods Branch, Islamic Azad University, Tehran, Iran

**Keywords:** bimodal AFM, optimization, soft matter, surface characterization, V-shaped cantilevers

## Abstract

As the application of atomic force microscopy (AFM) in soft matter characterization has expanded, the use of different types of cantilevers for these studies have also increased. One of the most common types of cantilevers used in soft matter imaging is V-shaped cantilevers due to their low normal spring constant. These types of cantilevers are also suitable for nanomanipulation due to their high lateral spring constants. The combination of low normal spring constant and high lateral spring constants makes V-shaped cantilevers promising candidates for imaging soft matter. Although these cantilevers are widely used in the field, there are no studies on the static and dynamic behavior of V-shaped cantilevers in multifrequency AFM due to their complex geometry. In this work, the static and dynamic properties of V-shaped cantilevers are studied while investigating their performance in multifrequency AFM (specifically bimodal AFM). By modeling the cantilevers based on Timoshenko beam theory, the geometrical dimensions such as length, base width, leg width and thickness are studied. By finding the static properties (mass, spring constants) and dynamic properties (resonance frequencies and quality factors) for different geometrical dimensions, the optimum V-shaped cantilever that can provide the maximum phase contrast in bimodal AFM between gold (Au) and polystyrene (PS) is found. Based on this study, it is found that as the length of the cantilever increases the 2nd eigenmode phase contrast decreases. However, the base width exhibits the opposite relationship. It is also found that the leg width does not have a monotone relationship similar to length and base width. The phase contrast increases for the range of 14 to 32 µm but decreases afterwards. The thickness of a V-shaped cantilever does not play a major role in defining the dynamics of the cantilever compared to other parameters. This work shows that in order to maximize the phase contrast, the ratio of second to first eigenmode frequencies should be minimized and be close to a whole number. Additionally, since V-shaped cantilevers are mostly used for soft matter imaging, lower frequency ratios dictate lower spring constant ratios, which can be advantageous due to lower forces applied to the surface by the tip given a sufficiently high first eigenmode frequency. Finally, two commercially available V-shaped cantilevers are theoretically and experimentally benchmarked with an optimum rectangular cantilever. Two sets of bimodal AFM experiments are carried out on Au-PS and PS-LDPE (polystyrene and low-density polyethylene) samples to verify the simulation results.

## Introduction

Since the invention of atomic force microscopy (AFM), different techniques have been introduced into the field to enhance and improve this nanotechnology equipment. In 2004, multifrequency AFM was introduced as a technique that can capture both topographical and material composition in a single-pass measurement [1]. In bimodal AFM, the first eigenmode is excited at or near the resonance frequency (reserved for topography measurements) while the second eigenmode is in open-loop capturing material composition via phase shift of the second eigenmode. Due to its unique capabilities, multifrequency AFM has gained the attention of different fields, such as, the measurement of nanoscale chemical and mechanical properties of human dentin [2], the mapping of viscoelastic materials [3], or the characterization of thin molecular films [4]. As different fields have implemented multifrequency AFM in their measurements and investigations, there is a need for further expansion of the field. One of the current limitation in the field is the theoretical understanding of V-shaped cantilevers in multifrequency AFM.

Microcantilevers are one of the most important components of micro-electromechanical systems (MEMS), superparamagnetic particle embedded microprobe (SPEM) sensors, or lab-on chips devices [5–8]. Microcantilevers are also an important component of atomic force microscopes (AFM). Due to their importance in AFM, there have been many studies on modeling the cantilevers to represent the dynamics of the cantilever more accurately. Wang et al. have used the finite element method (FEM) as an alternative approach to obtain the stiffness and the natural frequencies of for both rectangular and V-shaped cantilevers [9]. Additionally, other studies were done by Cleveland et al. [10] to measure the stiffness of AFM cantilevers. Later on, Sader et al. developed a nondestructive method for the evaluation of the spring constant, which relies solely on the determination of the unloaded resonant frequency of the cantilever [11]. This work was in contrast to the method of Cleveland et al., which required the attachment of masses to the cantilever to determine the spring constant. In recent years, cantilevers have provided valuable information by approximating the physical, mechanical, and chemical properties of samples. The performance of microbeams is determined by various parameters such as spring constant, quality factor, and resonant frequencies. Research has been conducted to improve AFM cantilevers in terms of manipulation, approximating material property, and images contrast. V-shaped and triangular cantilevers are widely employed in AFM imaging techniques due to their stability. Bushan stated that the cantilever stylus used in the AFM should have properties such as low normal spring constant, high resonance frequency, high quality factor, high lateral spring constant, and short cantilever length [12]. V-shaped cantilevers have a unique set of properties, that is, low normal stiffness and high lateral stiffness. Therefore, they can apply low forces to soft matter in normal mode while having a stable lateral mode. Additionally, their high lateral stiffness is advantageous in nanomanipulation applications moving particles over surfaces [12]. Morris stated in his book that researchers found that for biological or cell imaging, V-shape cantilevers are more appropriate than rectangular cantilevers due to higher torsional and lateral stability [13]. The higher stability of V-shaped cantilevers with a low normal spring constant provides a unique combination, which is not found in rectangular cantilevers.

There are two major applications of AFM that currently use V-shaped cantilevers. First, in static-mode AFM, that is, contact-mode AFM, V-shaped cantilevers are used in the modification of surfaces and the movement of nanoparticles to manufacture nanostructures [14–15]. In order to know the required force to move a particle on a surface, it is crucial to know the force applied by the cantilever to the surface. This is done by multiplying the deflection of the cantilever with its spring constant. Therefore, an accurate and reliable methodology to measure the spring constant is essential for successful manipulation by AFM and there are many studies focused on this subject [16–17]. It is known that the torsional spring constant of a cantilever should be minimized in order to make it more sensitive to forces [18]. It is also known that V-shaped cantilevers are more sensitive to lateral forces than rectangular cantilevers [19–20]. Many studies have been carried out order to find the spring constant of V-shaped cantilevers. For example, Albrecht et al. used the parallel beam method to approximate the spring constant of V-shaped cantilevers [21]. Afterwards, Butt and Sader introduced other models to calculate spring constants [22–23]. These methods were improved by Sader to a higher accuracy [11,24].

As a second category of application of V-shaped cantilevers, dynamic AFM is used to characterize soft matter. For example, Korayem et al. have carried out a free-vibration analysis of V-shaped AFM cantilevers the surfaces of which were covered by piezoelectric elements [25]. Sun et al. used an analytical method based on the Rayleigh–Ritz method to analyze the nonlinear vibrational behavior of single and double tapering cantilevers [26]. Ahmadi et al. have studied the vibrational behavior of rectangular and V-shaped AFM cantilevers using FEM [27].

All abovementioned researchers focused on the accuracy and sensitivity of the microscope by interpreting and analyzing the dynamic and vibration behavior. One way to increase accuracy and sensitivity in atomic force microscopy is exciting the higher modes of the cantilever (higher-modes AFM) or exciting multiple modes simultaneously (multifrequency AFM). It is important to simulate the dynamic and vibration behavior of the AFM under these conditions to interpret the dependency of sensitivity and material composition contrast on vibrational and dynamic properties. The ideal situation for achieving the best contrast in multifrequency or higher-mode AFM would be using AFM cantilevers with higher eigenmode frequencies equal or close to the higher harmonics [28–30]. This can be achieved by modifying the AFM cantilever designs. For example, it was shown that, by modifying the mass distribution and geometry of a rectangular cantilever, the ratio between the second eigenmode and the first eigenmode frequency decreases, which increases the chance of having self-excitation for higher harmonics [31–33]. By modifying the cantilever mass distribution this ratio can be brought closer to an integer value causing self-excitation [34–36]. Additionally, there are studies in which cross sections of the cantilever were altered [36–37] or hole structures were cut into the cantilevers [38–41] to achieve the same goal. Some researchers have studied the effect of geometry of rectangular cantilevers in bimodal AFM [42]. Also, the effect of the frequency ratio by considering a hole at different locations of the cantilever was studied. This led to the introduction of biharmonic AFM in the field [28].

This work focuses on using V-shaped cantilevers in multifrequency, specifically bimodal, AFM. The optimum geometrical dimensions of V-shaped cantilevers that can provide maximum phase contrast (material composition) are found by simulations. The effect of geometry on static and dynamic parameters of cantilevers such as mass, spring, and frequency of first and second eigenmodes is studied. Afterwards, a dynamic analysis based on bimodal AFM for V-shaped cantilevers is done to find the effect of length, base width, leg width, and thickness on vibrational analysis and, consequently, material composition contrast. After finding the optimum dimensions, two different commercial V-shaped cantilevers are compared by performing bimodal AFM on a polymer blend of polystyrene and low-density polyethylene (PS-LDPE). Additionally, two V-shaped cantilevers are compared with an optimum rectangular cantilever [42] which is a common choice of cantilevers in bimodal AFM. Finally, an analytical relationship is developed based on the numerical calculations. Based on this relationship, users can obtain a better understanding of expected phase contrast by providing the dimensions of the cantilever. This relationship can help experimentalists for selecting an appropriate cantilever.

## Theory

**Figure 1:**
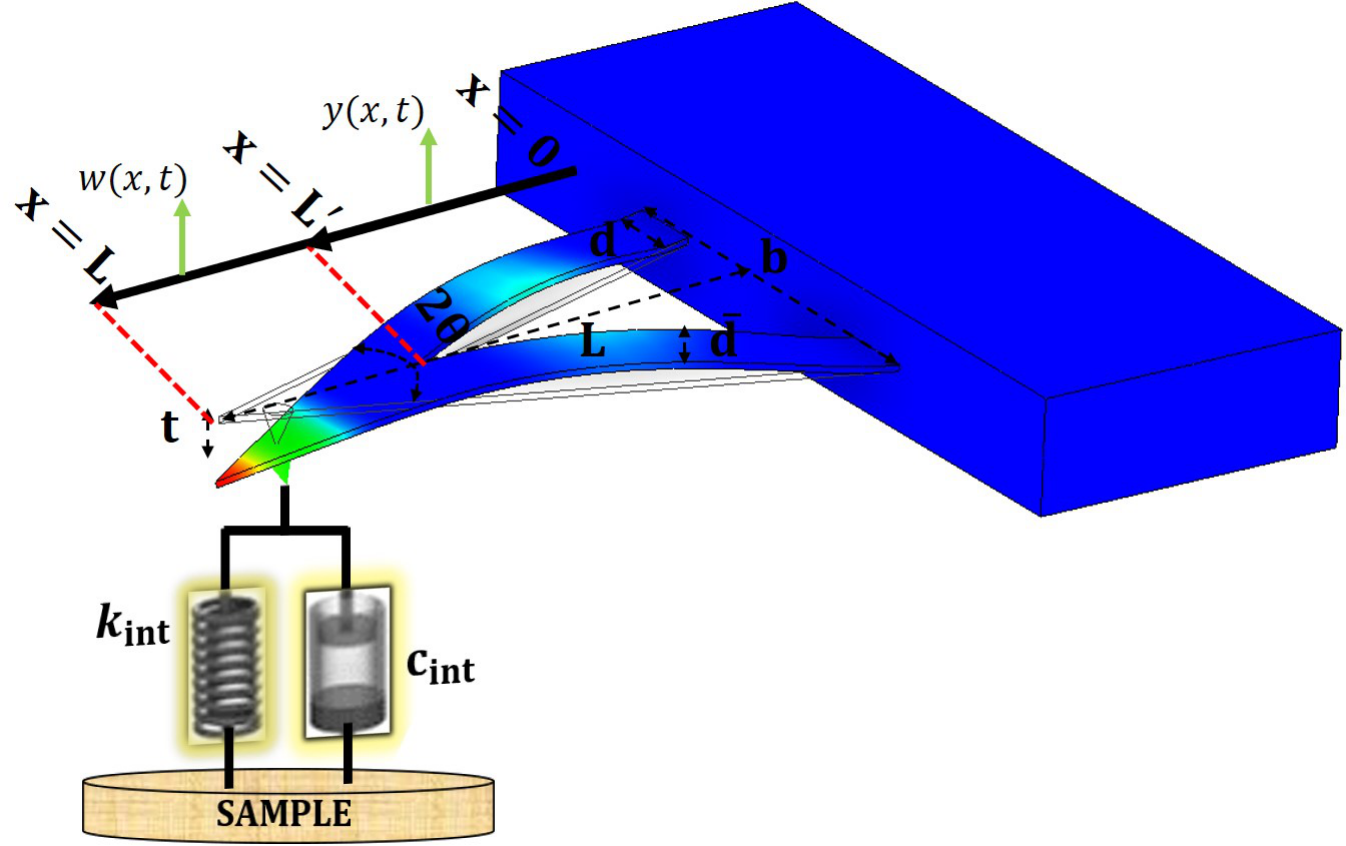
Schematic of V-shaped cantilevers with different geometrical parameters.

Unlike the uniform cross section of rectangular cantilevers throughout their length, the cross section of V-shaped cantilevers varies over the length. Therefore, in order to model them as Timoshenko’s beam, the equation of motion needs to be divided into two portions as shown in Figure 1. The first portion, shown in Equation 1, is the equation of motion related to the length of the cantilever base to the point where the two legs merge. For this range, the cross section is a rectangle on both sides:

[1] $$\begin{array}{l} \frac{\partial }{\partial x}\left[KGA_{1}\left(\frac{\partial y\left(x,t\right)}{\partial x}-\phi \left(x,t\right)\right)\right]-c\frac{\partial y\left(x,t\right)}{\partial t}\\ -\rho A_{1}\frac{\partial^{2}y\left(x,t\right)}{\partial t^{2}}=0, \end{array}$$

[2] $$\begin{array}{l} \frac{\partial }{\partial x}\left(EI_{1}\frac{\partial \phi \left(x,t\right)}{\partial x}\right)+KGA_{1}\left(\frac{\partial y\left(x,t\right)}{\partial x}-\phi \left(x,t\right)\right)\\ -\rho I_{1}\frac{\partial^{2}\phi \left(x,t\right)}{\partial t^{2}}=0, \end{array}$$

where 0 ≤ *x* ≤ (*L* – *L*′). In Equation 1 and Equation 2, *K*, *G*, *A*_1_, *y*(*x*,*t*), ϕ(*x*,*t*), ρ, *I*, *E* and *c* are shear coefficient, shear modulus, area of cross section, transverse deflection of the beam, bending angle of the beam, mass density of the beam, moment of inertia of cross section, Young’s modulus, and internal damping of the cantilevers, respectively. The cross-sectional area and moment of inertia shown in Equation 1 can be expressed as shown in Equation 3 and Equation 4, respectively:

[3] $$A_{1}=2dt,$$

[4] $$I_{1}=2\left(\frac{1}{12}\right)dt^{3}.$$

In Equation 5 and Equation 6, the equation of motion for the left side of the cantilever from the side where the legs merge to the tip of the cantilever is provided. Although the constants are the same, *w*(*x*,*t*) and θ(*x*,*t*) are the transverse deflection and bending angle of this part.

[5] $$\begin{array}{l} \frac{\partial }{\partial x}\left[KGA_{2}\left(\frac{\partial w\left(x,t\right)}{\partial x}-\theta \left(x,t\right)\right)\right]-c\frac{\partial w\left(x,t\right)}{\partial t}\\ -\rho A_{2}\frac{\partial^{2}w\left(x,t\right)}{\partial t^{2}}=0, \end{array}$$

[6] $$\begin{array}{l} \frac{\partial }{\partial x}\left(EI_{2}\frac{\partial \theta \left(x,t\right)}{\partial x}\right)+KGA_{2}\left(\frac{\partial w\left(x,t\right)}{\partial x}-\theta \left(x,t\right)\right)\\ -\rho I_{1}\frac{\partial^{2}\theta \left(x,t\right)}{\partial t^{2}}=0, \end{array}$$

where *L* – *L*′ ≤ *x* ≤ *L*. For this range of length, the cross section and moment of inertia for the above equations are denoted as *A*_2_ and *I*_2_ which are defined as shown in Equation 7 and Equation 8, respectively:

[7] $$A_{2}=2dt\left(1-r_{\text{b}}\eta_{1}\right),$$

[8] $$I_{2}=\frac{1}{12}2dt^{3}\left(1-r_{\text{b}}\eta_{1}\right),$$

where *r*_b_ is the width taper ratio for the left portion of the cantilever and η_1_ is the ratio that shows how far the location is from the merged end of the two arms. In other words, if the location is *x* = *L*, that is, at the tip of the cantilever, η_1_ is 1. These two quantities are defined in Equation 9 and Equation 10:

[9] $$r_{\text{b}}=1-\frac{b_{2}}{2d},$$

[10] $$\eta_{1}=\frac{x-\left(L-L^{\prime }\right)}{L^{\prime }}.$$

In this work, it is assumed the tip is at the free end of the cantilever and the cantilever is not parallel to the sample surface. The angle between the cantilever and the surface is assumed to be α. Therefore, the boundary conditions for the Timoshenko beam is as follows:

[11] y(0,t)=0 and ϕ(0,t)=0  B.C.'s at x=0,

[12] $$\begin{array}{l} y\left(L-L^{\prime },t\right)=w\left(\left(L-L^{\prime }\right),t\right)\text{ and }\\ \phi \left(\left(L-L^{\prime }\right),t\right)=\theta \left(\left(L-L^{\prime }\right),t\right)\text{ B}\text{.C}\text{.'s at }x=L-L^{\prime }. \end{array}$$

Equation 11 indicates the single clamped condition or zero transverse deflection and angle of bending at the clamped end of the beam. Equation 12 illustrates the continuity integration on deflection and slope at *x* = *L* – *L*′. The following equations (Equation 13) and (Equation 14) are the force balance (Σ*F*) and moment balance (Σ*M*), respectively:

[13] $$\begin{array}{l} -KGA_{2}\left(\frac{\partial w\left(L,t\right)}{\partial x}-\theta \left(L,t\right)\right)\\ =\left(K_{\text{n}}\cos^{2}\alpha +K_{\text{t}}\sin^{2}\alpha \right)w\left(L,t\right)\\ +l_{\text{tip}}\cos\alpha \sin\alpha \left(K_{\text{t}}-K_{\text{n}}\right)\theta \left(L,t\right), \end{array}$$

[14] $$\begin{array}{c} -EI_{2}\frac{\partial \theta \left(L,t\right)}{\partial x}=l_{\text{tip}}\cos\alpha \sin\alpha \left(K_{\text{t}}-K_{\text{n}}\right)w\left(L,t\right)\\ +l_{\text{tip}}^{2}\left(K_{\text{t}}\cos^{2}\alpha +K_{\text{n}}\sin^{2}\alpha \right)\theta \left(L,t\right). \end{array}$$

Since the cantilever is not parallel to the surface, there are horizontal and vertical conjugates of tip–sample force interactions. Therefore, the tip interactions are represented via both normal and tangential forces. Hence, the contact is described by normal (*K*_n_) and tangential (*K*_t_) linear springs, respectively.

Additionally, the governing equations of motion of the beam can be rewritten in modal form derived by FEM. These equations of motion (EOM) in matrix form can be defined as shown in Equation 15:

[15]
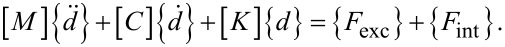


*F*_exc_(*t*) in bimodal AFM is the simultaneous excitation force of two fundamental cantilever resonant frequencies as *F*_exc_ = *F*_01_ cos ω_1_*t* + *F*_02_ cos ω_2_*t* where ω_1_ and ω_2_ are the first and second resonant frequencies. This form of EOM can express the internal damping coefficient as proportional damping model in terms of the mass and stiffness matrices. Therefore, the internal damping factor, which is written in Equation 1 through Equation 4 is found by Equation 16:

[16] $$C=a_{0}M+a_{1}K,$$

where *M* is the mass matrix, *K* is the stiffness matrix, *a*_0_ is the mass damping coefficient, and *a*_1_ is the stiffness damping coefficient. Based on the Rayleigh’s proportional damping shown in Equation 16, the modal damping factors can be written as:

[17] $$\gamma_{i}=\frac{a_{0}}{2\omega_{i}}+\frac{a_{1}\omega_{i}}{2}.$$

In Equation 17, subscript *i* represents the corresponding eigenmode. By finding γ*_i_*, the corresponding eigenmode quality factor can be found since: *Q_i_* = 1/2γ*_i_*. The quality factors of each eigenmode in addition to their corresponding resonant frequencies establish the diagonal damping matrix as shown in Equation 18:

[18] $$C_{\text{diagonal}}=\left[\begin{array}{cccc} \frac{\omega_{1}}{Q_{1}} & 0 & 0 & ...\\ 0 & \frac{\omega_{2}}{Q_{2}} & 0 & 0\\ 0 & 0 & \frac{\omega_{3}}{Q_{3}} & 0\\ ... & 0 & 0 & ... \end{array}\right].$$

Consequently, the damping matrix can be described as:

[19] $$C={\left(\Phi^{\text{T}}\right)}^{-1}C_{\text{diagonal}}\left(\Phi \right),$$

where Φ is the matrix of eigenvectors. Based on the above equations, the transverse deflection of the beam in bimodal AFM can be derived in the following form:

[20] $$y\left(x,t\right)=A_{1}\sin\left(\omega_{1}t+\phi_{1}\right)+A_{2}\sin\left(\omega_{2}t+\phi_{2}\right),$$

where *A*_1_, *A*_2_, ϕ_1_ and ϕ_2_ are the oscillation amplitude and phase response of the first and second bending eigenmodes, respectively.

## Results and Discussion

### Simulation analysis

Figure 1 represents the schematic of the V-shaped cantilever where the length (*L*), width (*b*), thickness (*t*), width of each leg (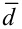), and the angle (2θ) are shown. We focused on finding the optimum for these geometrical dimensions of a V-shaped cantilever with the aim to enhance the signal-to-noise ratio (SNR) and, consequently, to enhance the second eigenmode phase signal.

A numerical study is carried out to model the V-shaped cantilevers given the abovementioned geometries while interacting with polystyrene (PS) and gold (Au) surfaces in bimodal AFM. The tip–sample force interactions are categorized into long-range van der Waals forces and short-range forces described by the Derjaguin, Muller and Toporov (DMT) model. The instantaneous tip distance *d* is defined as *d* = *z*_0_ + *z*_c_ + *z*_1_ + *z*_2_, where *z*_0_ and *z*_c_ are the average tip deflection and the average tip–surface separation, respectively. Depending on the value of *d*, tip–sample force interactions are modeled as:

[21] $$F_{\text{ts}}\left(d\right)=\{\begin{array}{c} F_{\text{vdW}}\left(d\right)=-\frac{H_{\text{air}}R}{6d^{2}}\\ F_{\text{DMT}}\left(d\right)=\frac{4E_{\text{eff}}\sqrt{R}}{3}{\left(h_{0}-d\right)}^{3/2}-\frac{H_{\text{air}}R}{6d^{2}} \end{array}.$$

Here *h*_0_ represents the intermolecular distance (0.165 nm), *H*_air_ is the Hamaker’s constant between tip and sample in air. *R* is the tip radius and *E*_eff_ is the effective elastic modulus between tip and sample. The material properties used in this simulation are shown in Table 1. The effects of length (*L*), overall width (*b*), the width of each leg (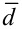) and the thickness (*t*) of the cantilever are optimized. In each round, the optimum parameters are selected and used for the next round of simulation for optimizing the other parameters. At the end, all parameters are optimized, providing the maximum phase contrast between Au and PS.

**Table 1:** Properties of Au and PS for AFM simulation.

*H*_tip–gold_ [10^−20^ J]	*E*(Au) [GPa]	*H*_tip–PS_ [10^−20^ J]	*E*(PS) [GPa]

12	75	7	3

**Figure 2:**
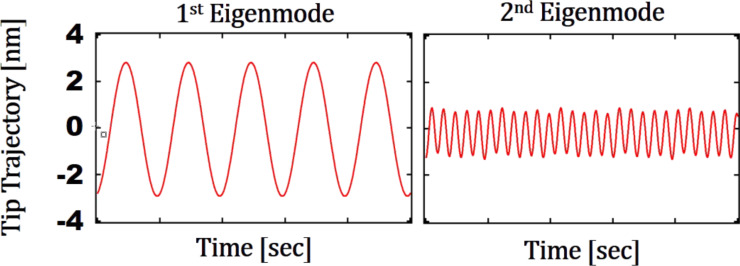
Tip trajectory of a V-shaped cantilever interacting with a Au sample (*L* = 85 µm, 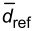 = 15 µm, *b*_ref_ = 86 µm, *t*_ref_ = 0.4 µm, *R*_tip_ = 9 nm, α = 0°) in time. Left: First eigenmode. Right: Second eigenmode. The setpoint is 60% for the first eigenmode amplitude.

In Figure 2, the tip trajectory of the first and second mode for the setpoint value of *Z*_c_ = 6 nm on Au is provided. In this round of simulations, the parameters were *L* = 85 µm, 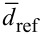 = 15 µm, *b*_ref_ = 86 µm, *t*_ref_ = 0.4 µm, *R*_tip_ = 9 nm, and α = 0° where α is defined as the angle between the cantilever and sample. Since this angle cannot be controlled by the user in AFM experiments, it is assumed that the cantilever is parallel to the surface for all simulations and experiments of this work. These results verify that the simulation can capture weakly perturbed oscillations of the V-shaped cantilevers in bimodal AFM. It should also be mentioned that the oscillation amplitudes for the first and second eigenmode are *A*_o1_ = 10 nm and *A*_o2_ = 0.4 nm for all simulations. These oscillation amplitudes, that is ratio and setpoint, were selected based on the results of the work of Gigler et al. who found the optimum oscillation amplitude ratios for imaging polymers in the repulsive regime of bimodal AFM [43]. Additionally, in order to have a better understanding of the dynamics of the AFM cantilever in this study, tip–sample force interactions on Au and PS samples are simulated and presented in Supporting Information File 1, Figure S1.

In order to study the effect of the length of the cantilever, all other geometrical dimensions are kept constant. However, it is important to note that as the length *L* changes, the static and dynamic properties of the cantilever also change. As shown in the Table 2, the dynamic spring constants, resonance frequencies, and quality factors for both first and second eigenmode change. These changes are calculated and implemented in the simulation for each round of modifications. The internal and external damping values *a*_0_ and *a*_1_ are also provided in the table.

**Table 2:** Range of lengths for V-shaped cantilevers with calculated properties.

Specifications of the V-shaped cantilever						

*E* = 150 GPa, ρ = 2320 kg/m^3^						
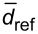 = 15 µm, *b*_ref_ = 86 µm, *t*_ref_ = 0.4 µm, *a*_0_ = 2 × 10^−10^, *a*_1_ = 3.7 × 10^3^						

*L* (µm)	*k*_1_ (N/m)	*k*_2_ (N/m)	*f*_1_ (kHz)	*f*_2_ (kHz)	*Q*_1_	*Q*_2_

85	0.1239	1.6148	96.82	515.35	161.29	558.69
90	0.10471	1.3862	86.825	464.7	145.18	540.31
95	8.9256 × 10^−2^	1.1982	78.28	421.06	131.3	518.95
105	6.6394 × 10^−2^	0.9123	64.58	350.32	108.74	471.60
115	5.0688 × 10^−2^	0.70973	54.15	295.79	91.39	423.40
125	3.9531 × 10^−2^	0.56202	46.03	252.85	77.84	378.11
135	3.1424 × 10^−2^	0.4524	39.59	218.55	67.03	337.04
145	2.5389 × 10^−2^	0.3695	34.42	190.75	58.30	300.84
155	2.0792 × 10^−2^	0.30522	30.18	167.85	51.13	269.12
165	1.7248 × 10^−2^	0.255	26.68	148.81	45.25	241.58
175	1.4459 × 10^−2^	0.21517	23.75	132.82	40.28	217.60
185	1.2243 × 10^−2^	0.18322	21.28	119.26	36.10	196.76
195	1.0456 × 10^−2^	0.15719	19.17	107.65	32.44	178.61

**Figure 3:**
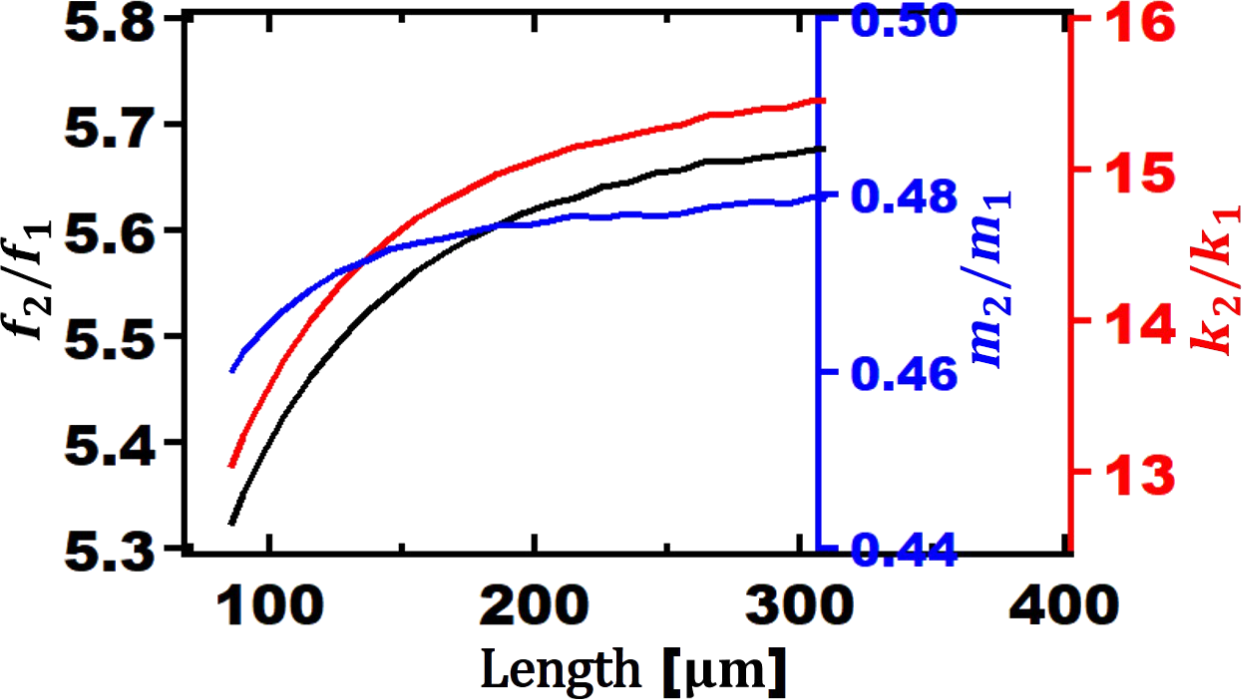
Ratio of second eigenmode to first eigenmode as a function of the length of V-shaped cantilevers for: frequency (black axis), mass (blue axis), and stiffness (red axis). The other geometrical parameters are: 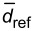 = 15 µm, *b*_ref_ = 86 µm, and *t*_ref_ = 0.4 µm.

Before observing the phase difference between Au and PS as a function of the length of the cantilever, it is important to find the trend of frequencies, stiffnesses and masses of the cantilever for the second and the first eigenmode of V-shaped cantilevers. For rectangular cantilevers, the ratio between the dynamic spring constant of the second eigenmode and that of the first eigenmode is a constant value of *k*_2_/*k*_1_ = 39. The frequency ratio is *f*_2_/*f*_1_ ≈ 6.27. Additionally, the mass ratio for rectangular cantilevers is also constant and is equal to *m*_2_/*m*_1_ = 1 = *m*/4. As shown in Figure 3, these ratios are not constant for V-shaped cantilevers. The ratio of frequency varies from 5.3 to 5.7 depending on the length of the cantilever. Similarly, ratios of masses and stiffnesses change. This can be an advantageous. In bimodal AFM with rectangular cantilevers, the tip–sample force interactions are dictated by the ratio of *k_i_A_i_* as it was previously shown by Ebeling and co-workers [44]. In other words, regardless of the dimensions of the rectangular cantilever selected, the user has can only control the sensitivity of the cantilever to tip–sample force interactions via the oscillation amplitude. In contrast, for V-shaped cantilevers, based on the length of the cantilever, the tip–sample force interactions can be different in bimodal AFM. This provides more flexibility during experiments.

In Figure 3, the ratio of the second to first eigenmode frequencies is given as a function of the cantilever length. It is well known that for rectangular cantilevers *f*_2_/*f*_1_ = 6.27, regardless of their dimensions. For V-shaped cantilevers the frequency ratio is different for different lengths. As the length of the cantilever increases, the frequency ratio increases. For the smallest length (85 µm), the frequency ratio is 5.32. Since this number is close to the whole number 5 and makes thus the second frequency almost a multiple integer of the first, the phase contrast is increased. It should be mentioned that these results suggest self-excitation capability for V-shaped cantilevers since the frequency ratios are smaller. This phenomenon is more likely to occur in liquid environment where the quality factor (*Q*) is lower than in air.

Unlike rectangular cantilevers where the ratio of dynamic spring stiffness is constant at 39.3, the ratio of a V-shaped cantilever *k*_2_/*k*_1_ shows a range from 13 to 15.5 N/m. This is another advantage of using V-shaped cantilevers in bimodal AFM due to the lower spring constant of the higher eigenmodes. Lower dynamic spring constant ratios for V-shape cantilevers suggest lower forces applied to surfaces. Considering the major application of V-shape cantilevers, this can be an additional reason for using V-shaped cantilever in bimodal AFM for characterizing soft matters such as polymers or biological samples. However, this advantage comes with a trade-off of having a lower first eigenmode frequency, which can lead to challenges in imaging soft matter as discussed by Nikfarjam and co-workers [45].

**Figure 4:**
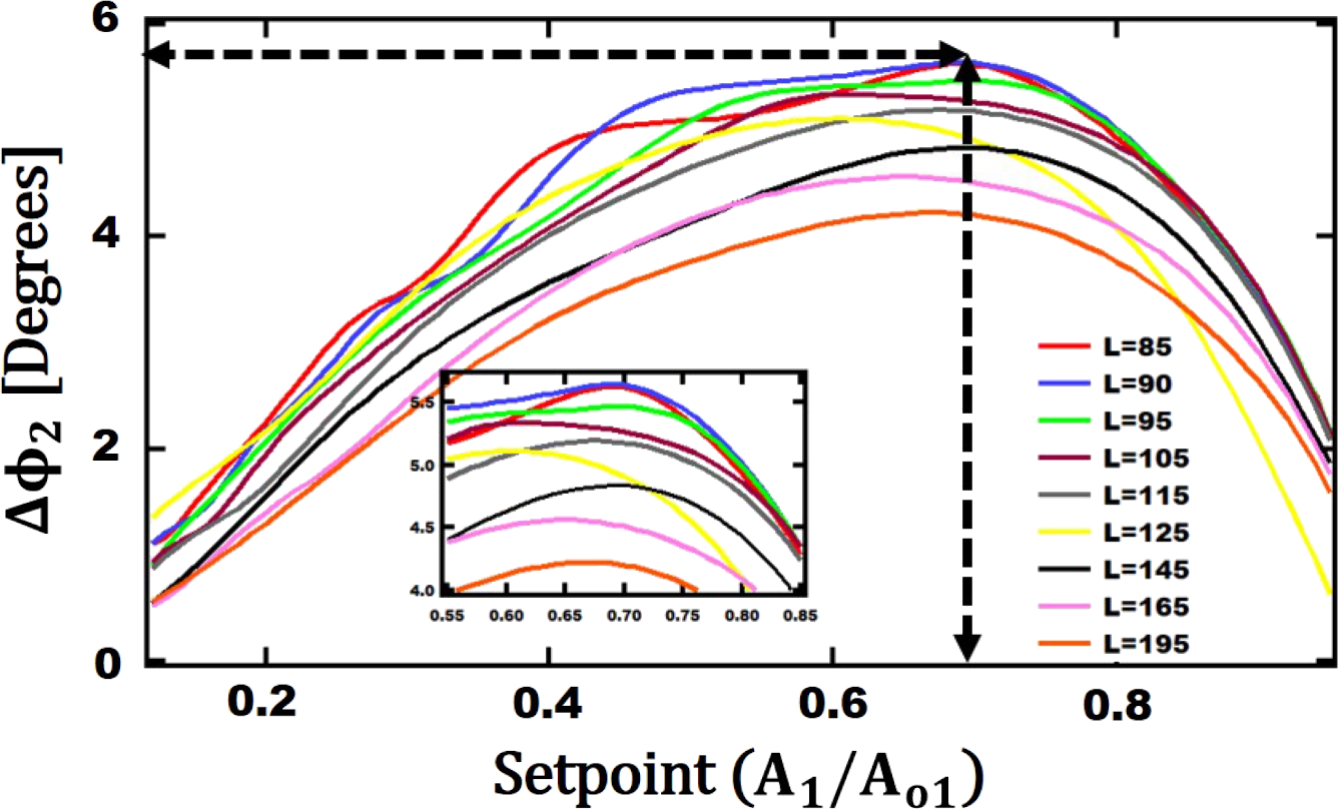
Second eigenmode phase difference between Au and PS versus setpoint for a given cantilever length. The other geometrical parameters are: 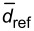 = 15 µm, *b*_ref_ = 86 µm, *t*_ref_ = 0.4 µm, *R*_tip_ = 9 nm, and α = 0°.

In Figure 4, the second eigenmode phase difference as a function of the first eigenmode setpoint is shown for Au and PS samples for different cantilever lengths. The first and second free oscillation amplitudes are *A*_o1_ = 10 nm and *A*_o2_ = 0.04 nm, respectively. It should be noted for all setpoints and cantilever lengths for both Au and PS the phase value is above 90° referring to the attractive regime. For a given V-shaped cantilever with 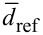 = 15 µm, *b*_ref_ = 86 µm, *t*_ref_ = 0.4 µm, *R*_tip_ = 9 nm, α = 0°, its length *L* has been varied in a range of 85 to 310 µm. After *L* = 195 µm there is a continuous decrease in phase contrast for longer lengths. Thus, results for *L* > 195 µm are not shown. Between 90 and 310 µm, the phase contrast between Au and PS decreases. The maximum phase contrast of 5.64° is observed at 90 µm when the setpoint for the first eigenmode amplitude is 69%. This is shown with horizontal and vertical dashed-arrows in Figure 4.

After optimizing the length of the cantilever, the next round of simulations is focused on the width *b*. For this set of simulations, the following geometrical parameters are set: *L*_opt_ = 90 µm, 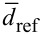 = 15 µm, and *t*_ref_ = 0.4 µm. The width of cantilever *b* was changed from 74 to 254 µm with 10 µm increments. By changing the width of cantilever *b*, the effective frequencies, stiffnesses and quality factors for the first and second eigenmode change. These values are derived and provided in Table 3.

**Table 3:** Range of base widths (*b*) for V-shaped cantilevers with calculated properties.

Specifications of the V-shaped cantilever						

*E* = 150 GPa, ρ = 2320 kg/m^3^						
*L*_opt_ = 90 µm, 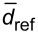 = 15 µm, *t*_ref_ = 0.4 µm, *a*_0_ = 2 × 10^−10^, *a*_1_ = 3.7 × 10^3^						

*b* (µm)	*k*_1_ (N/m)	*k*_2_ (N/m)	*f*_1_ (kHz)	*f*_2_ (kHz)	*Q*_1_	*Q*_2_

74	0.1058	1.3113	93.392	489.55	155.72	550.10
84	0.10493	1.3764	87.822	468.76	146.81	542.05
94	0.10363	1.4153	83.081	448.54	139.14	533.10
104	0.10198	1.4339	78.887	428.9	132.34	523.33
114	0.10003	1.4374	75.079	409.87	126.16	512.88
124	9.7842 × 10^−2^	1.4297	71.572	391.53	120.47	501.65
134	9.5437 × 10^−2^	1.4137	68.294	373.88	115.07	489.82
144	9.2852 × 10^−2^	1.3915	65.205	356.94	110.09	477.87
154	9.0133 × 10^−2^	1.3648	62.284	340.71	105.23	464.92
164	8.7294 × 10^−2^	1.3349	59.504	325.18	100.61	451.83
174	8.4388 × 10^−2^	1.3029	56.863	310.38	96.25	438.71
184	8.1419 × 10^−2^	1.2695	54.342	296.25	91.12	425.57
194	7.8555 × 10^−2^	1.2386	51.993	283.24	88.15	412.53
204	7.5684 × 10^−2^	1.2056	49.731	270.54	84.31	399.12
214	7.2861 × 10^−2^	1.1722	47.613	258.67	80.684	386.20
224	6.974 × 10^−2^	1.1372	45.472	246.88	76.765	370.48
234	6.691 × 10^−2^	1.1049	43.516	236.04	73.450	357.59
244	6.4051 × 10^−2^	1.0713	41.638	225.68	70.31	345.03
254	6.1499 × 10^−2^	1.0395	39.908	215.94	67.321	332.8

**Figure 5:**
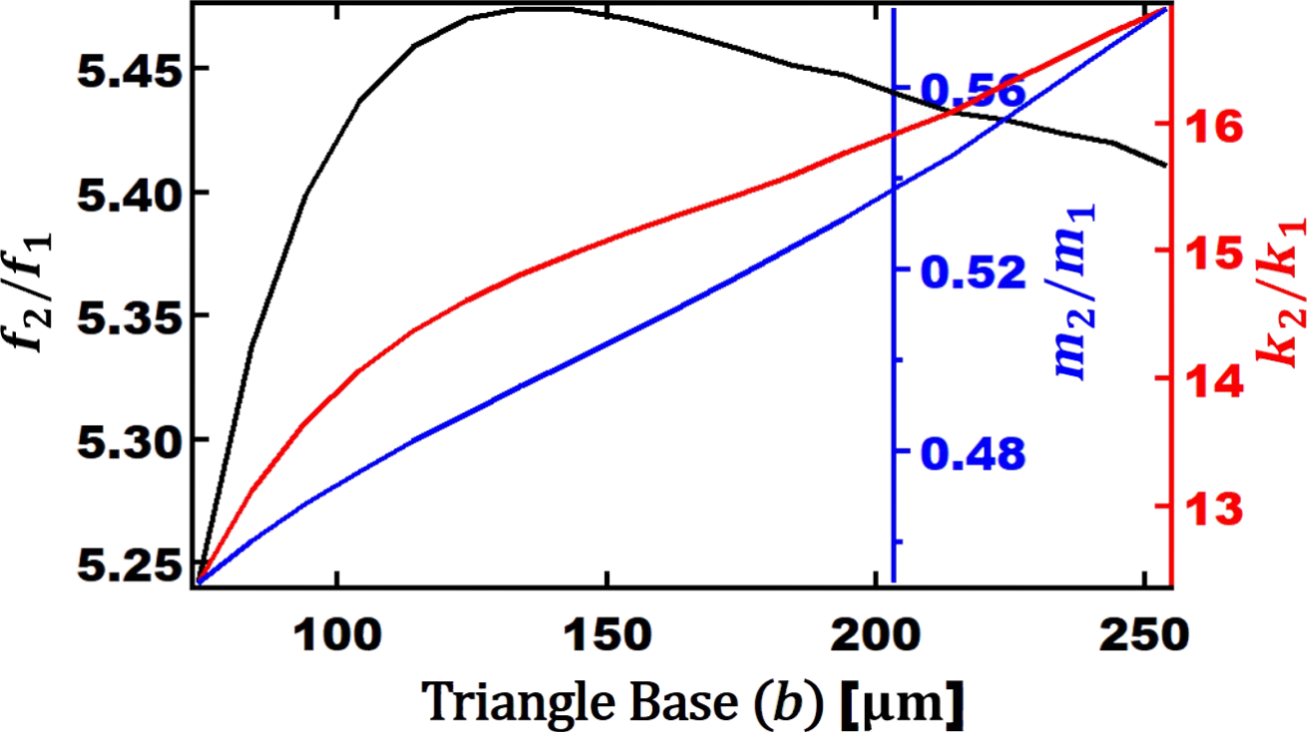
Ratio of second eigenmode to first eigenmode as a function of the triangle base width (*b*) of V-shaped cantilevers for: frequency (black axis), mass (blue axis), and stiffness (red axis). The other geometrical parameters are: *L*_opt_ = 90 µm, 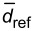 = 15 µm, and *t*_ref_ = 0.4 µm.

In Figure 5, the frequency, mass, and stiffness ratios of first and second eigenmode are presented. The interval of frequency ratio is 5.2 to 5.5. The value of *b* has a smaller effect on the frequency ratio than the length. The stiffness ratio changes from 12.4 to 17. The ratio between the masses for the V-shaped cantilevers is not constant and varies from 0.45 to 0.58 depending on the width of the cantilever. As mentioned in the discussion of Figure 3, these variable ratios of higher eigenmodes to the fundamental one can be crucial in selecting and designing the optimum cantilever for bimodal AFM imaging.

**Figure 6:**
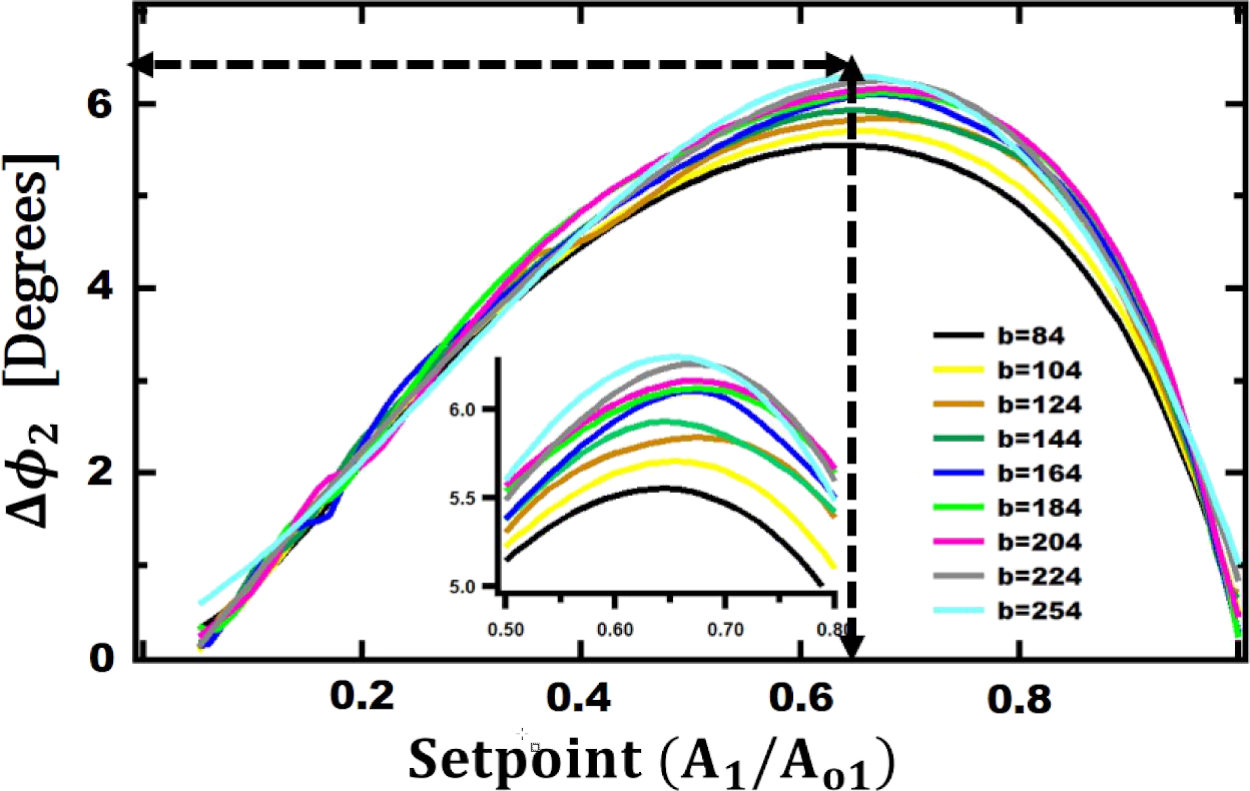
Second eigenmode phase difference between Au and PS as a function of the setpoint for a given cantilever base width. The other geometrical parameters are: *L*_opt_ = 90 µm, 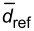 = 15 µm, and *t*_ref_ = 0.4 µm.

After finding the relationship between parameters of higher eigenmodes and those of the fundamental one, we have studied the effect of the width *b* on the maximum phase contrast observed between Au and PS while changing *b* from 74 to 254 µm. As shown in Figure 6, the maximum contrast is observed when *b* = 254 µm and the setpoint is 65%. The maximum contrast is 6.3° at optimum length (*L*) and width (*b*).

So far, we have optimized the length and base width of V-shaped cantilevers. With these two optimum parameters, a new set of simulation is carried out to find the width of each leg of cantilever that can provide enhanced phase contrast. Similarly, the stiffness, frequency and quality factors of the first and second eigenmodes are found and tabulated in Table 4. As shown in the table, the width of each leg 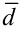 is between 14 and 40 µm.

**Table 4:** Range of each leg’s widths (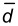) for V-shaped cantilevers with calculated properties.

Specifications of the V-shaped cantilever						

*E* = 150 GPa, ρ = 2320 kg/m^3^						
*L*_opt_ = 90 µm, *b*_opt_ = 254 µm, *t*_ref_ = 0.4 µm, *a*_0_ = 2 × 10^−10^, *a*_1_ = 3.7 × 10^3^						

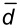 (µm)	*k*_1_ (N/m)	*k*_2_ (N/m)	*f*_1_ (kHz)	*f*_2_ (kHz)	*Q*_1_	*Q*_2_

14	5.6172 × 10^−2^	0.98543	39.005	211.43	66.40	331.1
15	6.1499 × 10^−2^	1.0395	39.908	215.94	67.321	332.8
20	8.9697 × 10^−2^	1.2663	44.589	239.07	75.70	364
25	0.12222	1.4319	50.002	262.11	84.69	389.73
28	0.14282	1.5162	53.411	274.12	90.86	405.71
30	0.15878	1.5966	56.245	283.83	94.79	412.11
32	0.17202	1.6683	58.348	289.62	99.27	421.74
35	0.19707	1.8473	62.812	302.88	106	431.84
40	0.23804	2.2568	70.229	325.0	118.28	453.02

**Figure 7:**
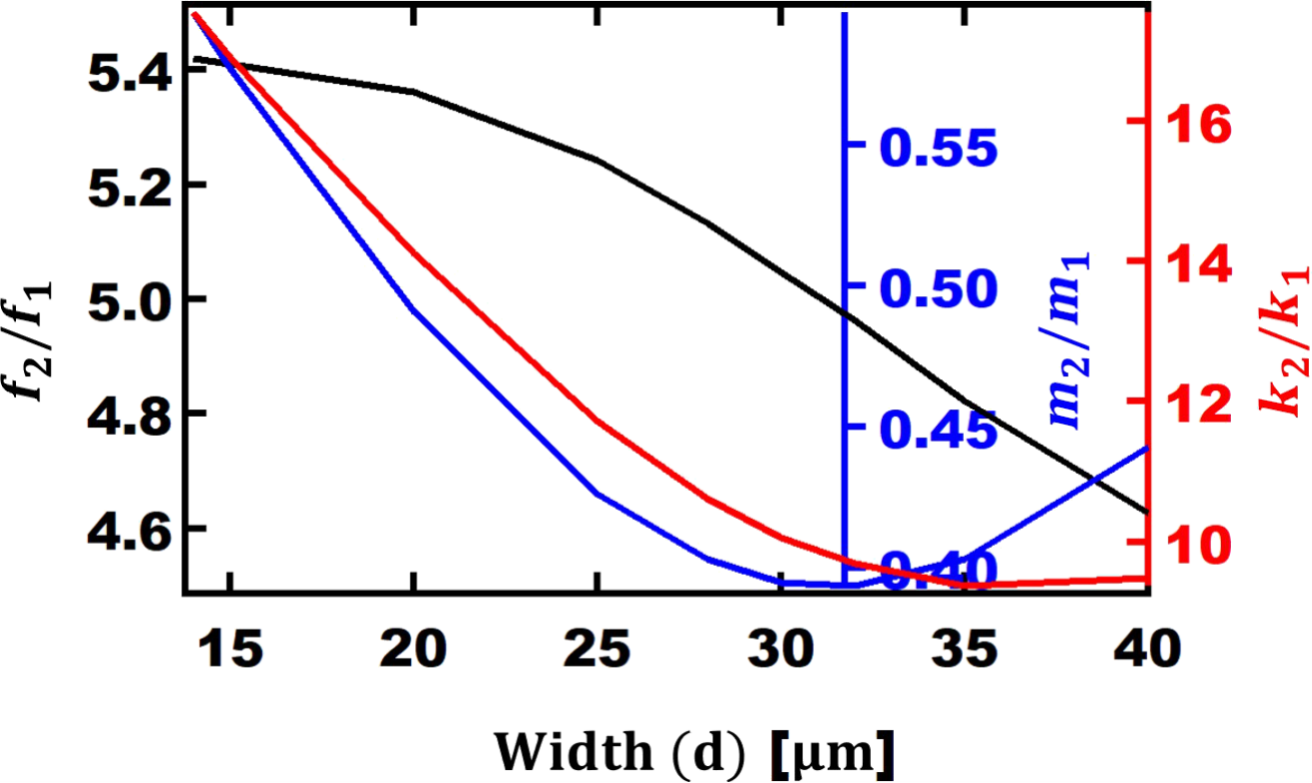
Ratio of second eigenmode to first eigenmode versus each arm’s width (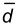) of V-shaped cantilevers for: frequency (black axis), mass (blue axis), and stiffness (red axis). The other geometrical parameters are: *L*_opt_ = 90 µm, *b*_opt_ = 254 µm, and *t*_ref_ = 0.4 µm.

In Figure 7, the frequency, the stiffness, and the mass ratio of second to first eigenmode as function of the width are shown. The frequency ratio is between 4.6 and 5.3, which is much lower than that of a rectangular cantilever. Although a reduction in stiffness is advantageous, a reduction in frequency might be disadvantageous for cases where the first eigenmode frequency is too low for imaging polymers. However, if the reduction in frequency ratio is due to a lower second eigenmode compared to the first eigenmode and the first eigenmode is sufficiently high, V-shaped cantilevers can be potentially a better selection for bimodal AFM imaging. Additionally, it is found that for 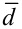 values near 30 µm, the frequency ratio is 5.04 which is close to the whole number 5. This can be advantageous since it was shown that when the second eigenmode is closer to a multiple integer of the fundamental eigenmode frequency, the phase signal of the higher eigenmodes is enhanced [28]. Additionally, a higher eigenmode frequency that is a multiple integer of the first one can help with providing more regular taps on surfaces. This can be useful in lower-quality environments where the concept of a “of forest of peaks” is seen [46]. As shown on the red axis of the Figure 7, the ratio between the stiffness values ranges from 9.3 to 17.5 and the ratio of masses changes from 0.39 to 0.6.

**Figure 8:**
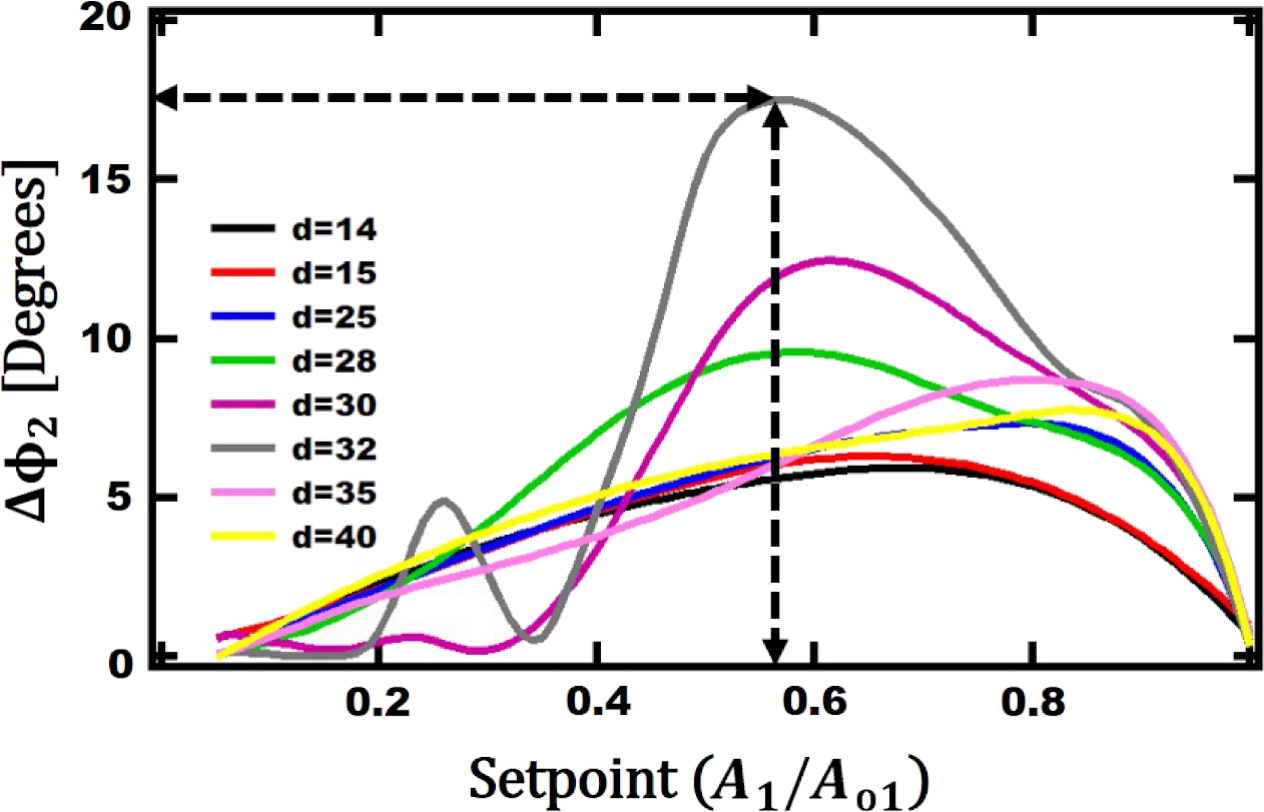
Phase difference of the Second eigenmode between Au and PS as a function of the setpoint for a given cantilever leg width length. The other geometrical parameters are: *L*_opt_ = 90 µm, *b*_opt_ = 254 µm, and *t*_ref_ = 0.4 µm.

In order to find the optimum width for each leg of V-shaped cantilever, the phase values on Au and PS have been recorded. The difference between the phase values is the expected phase contrast. Figure 8 shows the phase difference between Au and PS for different 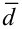 values. For 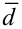 = 32 µm, the phase contrast increases up to 17° for a setpoint around 57%. By increasing the setpoint, the phase contrast decreases again.

The last parameter to optimize is the thickness (*t*). So far, it is found that with *L*_opt_ = 90 µm, *b*_opt_ = 254 µm, and 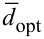 = 32 µm, the phase contrast is enhanced on the second eigenmode. With these parameters, the thickness is optimized accordingly as shown in Table 5.

**Table 5:** Range of each cantilever thicknesses for V-shaped cantilevers with calculated dynamic properties.

Specifications of the V-shaped cantilever						

*E* = 150 GPa, ρ = 2320 kg/m^3^						
*L*_opt_ = 90 µm, *b*_opt_ = 254 µm, 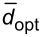 = 32 µm						

*t* (µm)	*k*_1_ (N/m)	*k*_2_ (N/m)	*f*_1_ (kHz)	*f*_2_ (kHz)	*Q*_1_	*Q*_2_

0.3	7.2845 × 10^−2^	0.70666	43.854	217.78	73.99	335.31
0.35	0.11559	1.121	51.142	253.92	86.09	378.25
0.4	0.17202	1.6683	58.348	289.62	99.27	421.74
0.45	0.24529	2.3782	65.705	326.12	110.29	450.63
0.5	0.3362	3.2594	72.981	362.17	122.29	480.04
0.55	0.44717	4.3347	80.252	398.2	134.14	504.77
0.6	0.58012	5.6231	87.520	434.2	145.91	525.40

**Figure 9:**
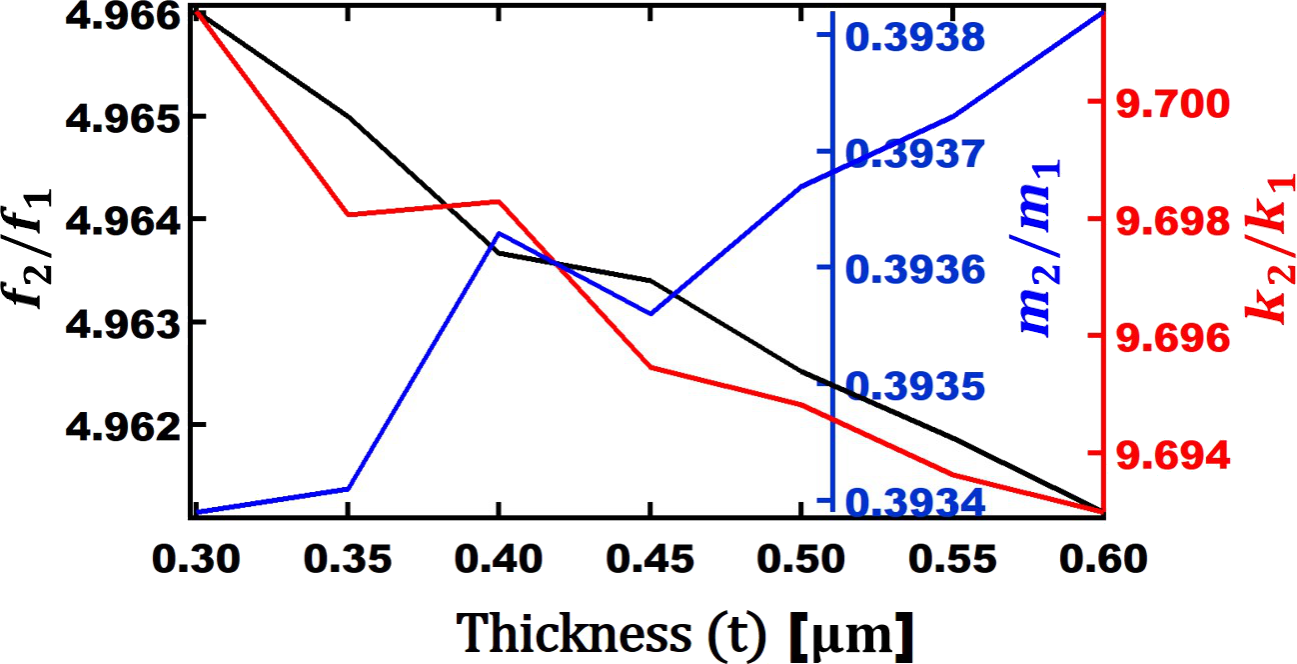
Ratio of second eigenmode to first eigenmode versus thickness (*t*) of V-shaped cantilevers for: frequency (black axis), mass (blue axis), and stiffness (red axis). The other geometrical parameters*: L*_opt_ = 90 µm, *b*_opt_ = 254 µm, and 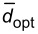 = 32 µm.

The above results are graphed in Figure 9 to represent the ratios of the above parameters. It is shown that thickness does not influence the frequency significantly. As shown, the frequency ratio changes from 4.962 to 4.966 for the whole range of thickness values from 0.3 to 0.6 µm. Figure 9 also provides some insight about which thickness can provide a higher phase contrast. A ratio of frequencies closest to the harmonic of the first eigenmode (i.e., *f*_2_/*f*_1_ closest to a whole integer) provides better phase contrast. When thickness is closer to 0.3 µm, the algebraic value of |*n* − *f*_2_/*f*_1_| is minimized (*n* = 5). Additionally, we observe small variations in the ratio of stiffnesses and masses. Overall, the thickness hardly influences the ratio of frequencies, stiffnesses and masses.

**Figure 10:**
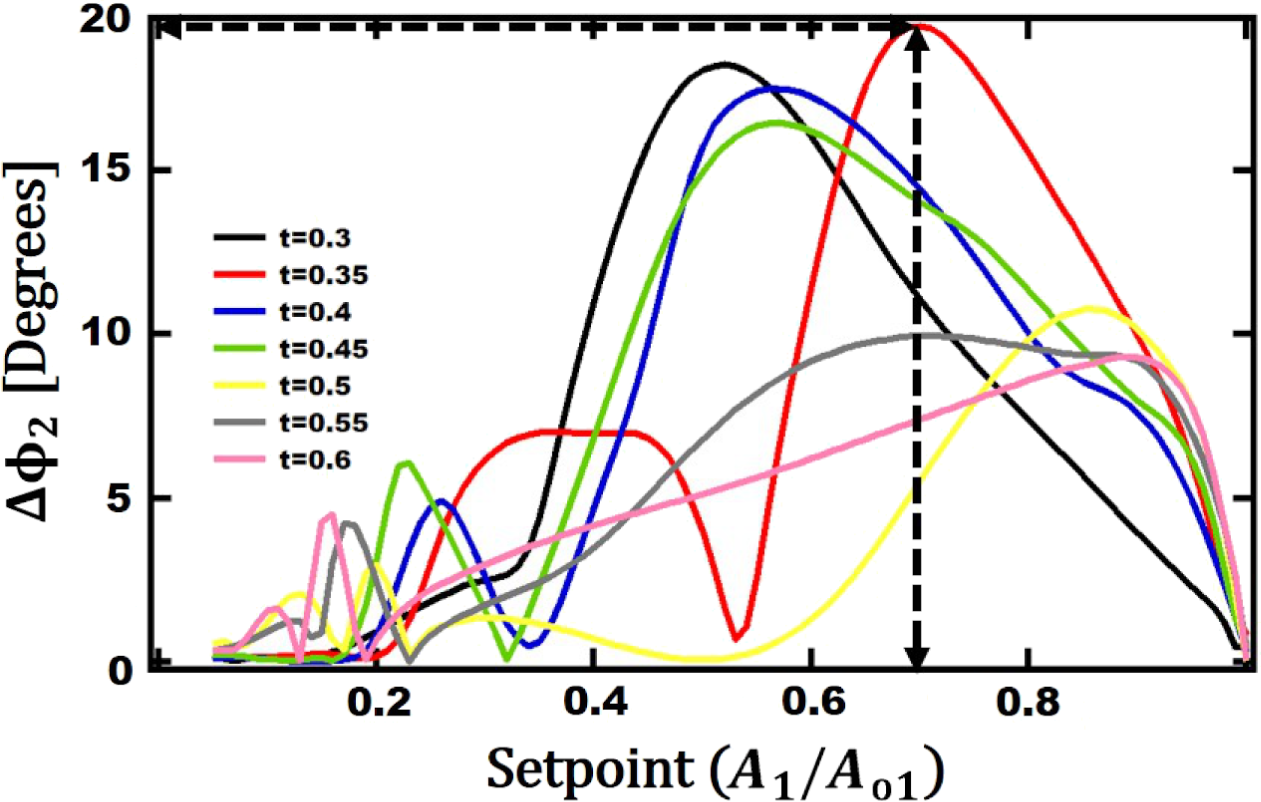
Second eigenmode phase difference between Au and PS as function of the setpoint for different cantilever thickness values. The other geometrical parameters are: *L*_opt_ = 90 µm, *b*_opt_ = 254 µm, and 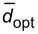 = 32 µm.

The effect of thickness on the phase contrast for different setpoints of the first eigenmode amplitude is shown in Figure 10. The maximum phase contrast of 19.4° is observed around a setpoint of 70% when the thickness is 0.35 µm. After this final round of simulations, the optimum geometrical dimensions of a V-shaped cantilever that can provide maximum phase contrast in bimodal AFM is found to be *L*_opt_ = 90 µm, *b*_opt_ = 254 µm, 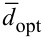 = 32 µm, and *t*_opt_ = 0.35 µm. In order to verify these results further, a bimodal AFM experiment is performed.

### Experimental analysis

In a recent study, the same optimization method is used to find the optimum rectangular cantilever [42]. Based on this study, a MULTI75G cantilever was selected as a commercially available probe that can provide maximum phase contrast in bimodal AFM studies. After finding the optimum dimensions for a V-shaped cantilever that can provide maximum phase contrast on the second eigenmode, two different types of commercially available V-shaped cantilevers are compared with the MULTI75G cantilever. Table 6 provides the specification of these cantilevers.

**Table 6:** Geometrical specifications provided by manufacturer.

model	length [µm]	base width [µm]	leg width [µm]	thickness [µm]

theoretically optimized	90	254	32	0.35
OMCL-TR400S	100	106	13.4	0.4
OMCL-TR400L	200	166	27.9	0.4
MULTI75G-R10	225	28	N/A	3

**Figure 11:**
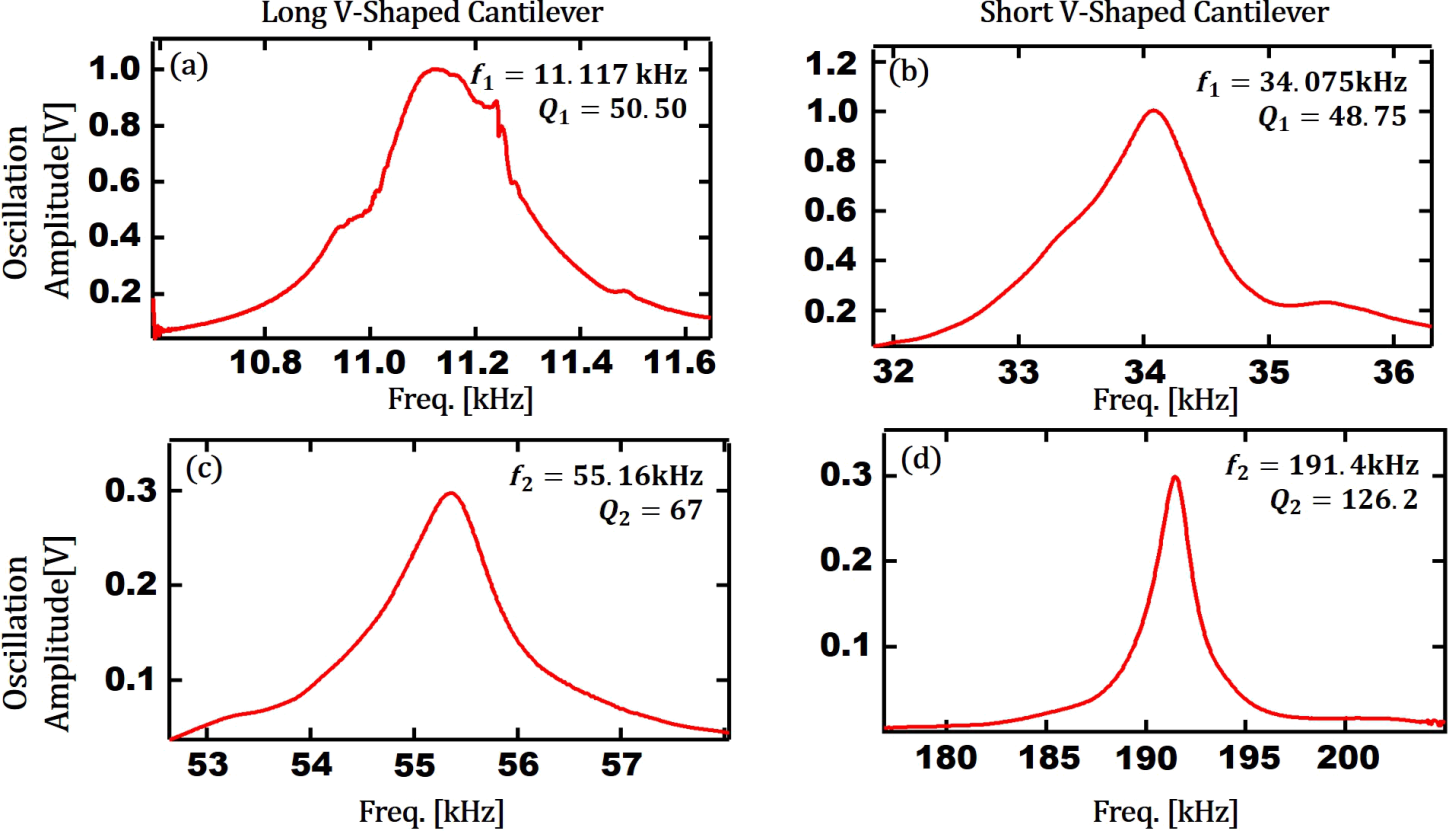
Amplitude versus frequency response: (a) 1st eigenmode frequency tune curve of the long V-shaped cantilever, (b) 1st eigenmode frequency tune curve of the short V-shaped cantilever, (c) 2nd eigenmode frequency tune curve of the long V-shaped cantilever and (d) 2nd eigenmode frequency tune curve of the short V-shaped cantilever.

Before performing bimodal AFM imaging, another simulation study using the abovementioned numerical code was carried out to compare the theoretical response of the above three models of cantilevers while interacting with Au and PS. In order to accurately calculate the resonance frequency and quality factors of each eigenmode for the V-shaped cantilevers, their frequency response curves are captured experimentally. By performing frequency sweep curves experimentally for both of the V-shaped cantilevers, the first and second eigenmode amplitude versus frequency responses of each V-shaped cantilever are measured as shown in Figure 11. It is known that the quality factor (*Q*) can be found by the ratio of *f*_res_/Δ*f* where Δ*f* is the resonance width at half-maximum.

**Figure 12:**
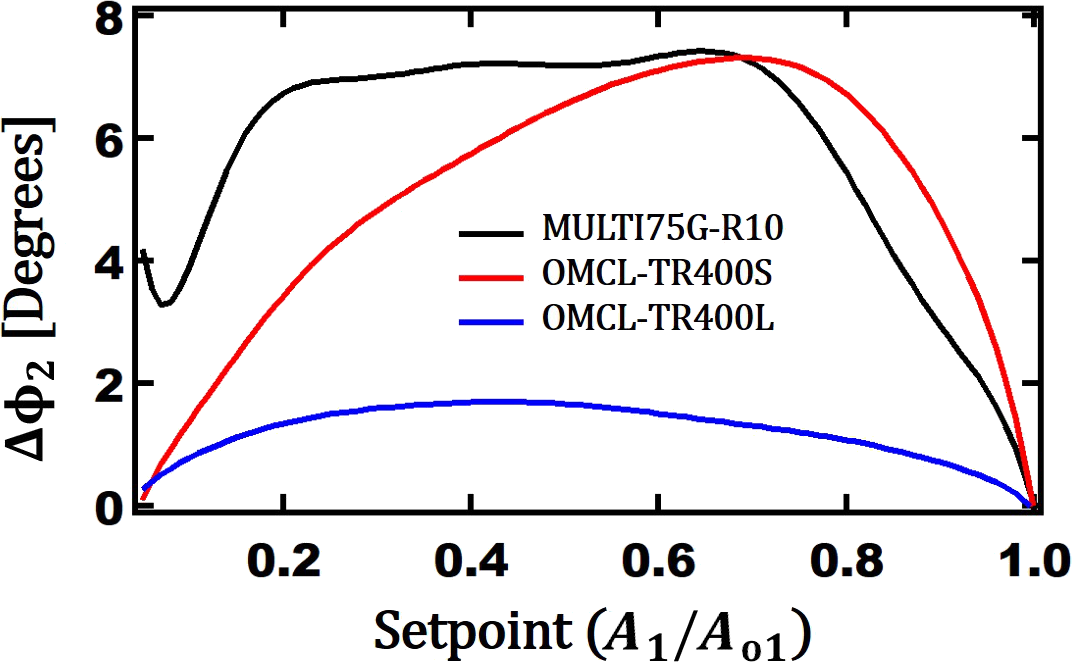
Simulation results of three commercial cantilevers in bimodal AFM done on Au and PS polymers. Vertical axis: the second eigenmode phase difference between Au and PS. Horizontal axis: first eigenmode setpoint. Black: MULTI75G, red: TR400Short, and blue: TR400Long.

Based on the simulation comparing these three cantilevers, the phase difference between Au and PS for the second eigenmode of each cantilever is plotted as a function of the first eigenmode setpoint. This result is presented in Figure 12. It is clear that the long V-shaped cantilever has the lowest phase contrast. However, the short V-shape can provide equal or higher phase contrast for a setpoint of 75% and above compared to the MULTI75G rectangular cantilever. It is important to mention that the V-shaped cantilevers have lower spring constants (*k*), which can apply a lower force to surfaces and minimize surface damage. This can be advantageous especially while imaging using higher eigenmodes or multifrequency AFM, as long as the first eigenmode frequency sufficiently high to perform soft matter imaging. Although the commercially used V-shaped cantilevers are not very close in dimensions to the theoretically optimum dimensions, one of them provide a phase contrast similar to that of the optimum rectangular cantilever.

These three cantilevers were used in an experimental bimodal AFM study. An Asylum Research MFP3D Origin atomic force microscope equipped with an ARC2 controller is used to perform the experiments. There are two sets of experiments done. Firstly, a PS polymer solution (molecular weight of 35000 dissolved in tetrahydrofuran (THF)) was spin-coated at 3500 rpm for 60 s on a cleaned Au substrate. All items were purchased from Sigma-Aldrich. After drying the sample, a portion of the PS thin film was scratched to expose the Au surface. The second sample is a polymer blend of PS and low-density polyethylene (LDPE) (HarmoniX sample purchased from Bruker) in order to challenge the cantilevers with more similar material properties.

**Figure 13:**
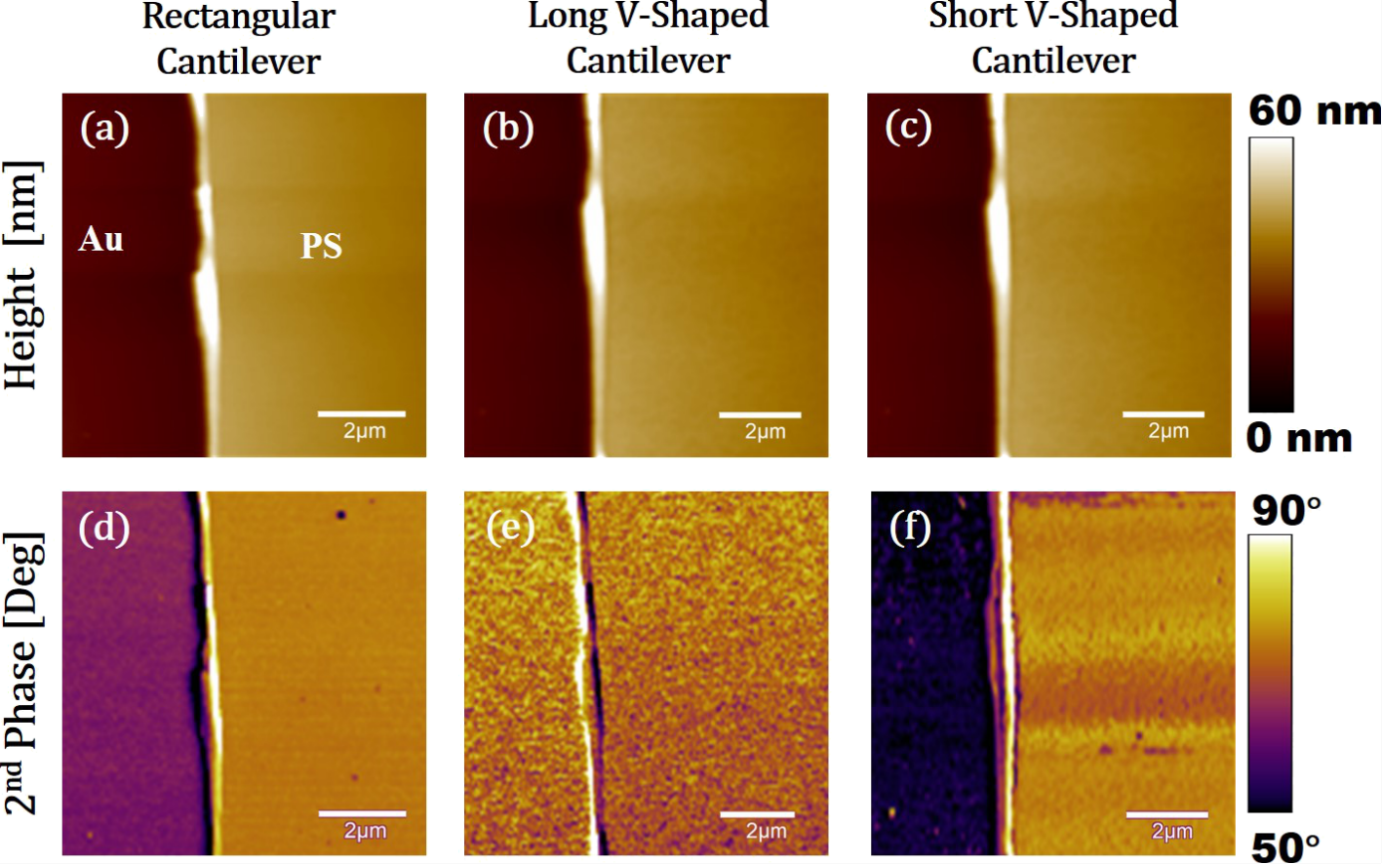
Bimodal AFM images of Au–PS samples. Left column: MULTI75G, middle column: TR400Long, right column: TR400Short. (a), (b), and (c) are height images. (d), (e), and (f) are phase images. All images were recorded at the same spot with a scan rate of 0.5 Hz, a setpoint ratio of 65%, a first eigenmode amplitude of *A*_1_ = 10 nm, and *A*_2_ = 2 nm. The gain was adjusted to maintain the quality of the images. The image size is 10 µm times 10 µm.

Figure 13a–c shows height or topography images of the Au–PS sample imaged in bimodal AFM using a rectangular, a long V-shaped, and a short V-shaped cantilever, respectively. The expected theoretical phase values are around 7° for 60% setpoint for the rectangular and the short V-shaped cantilever and around 2° for the long V-shaped cantilever. These results are also observed in Figure 13. Although Figure 13a–c shows relatively similar topographies, Figure 13f show the highest phase contrast between Au and PS for the short V-shaped cantilever.

**Figure 14:**
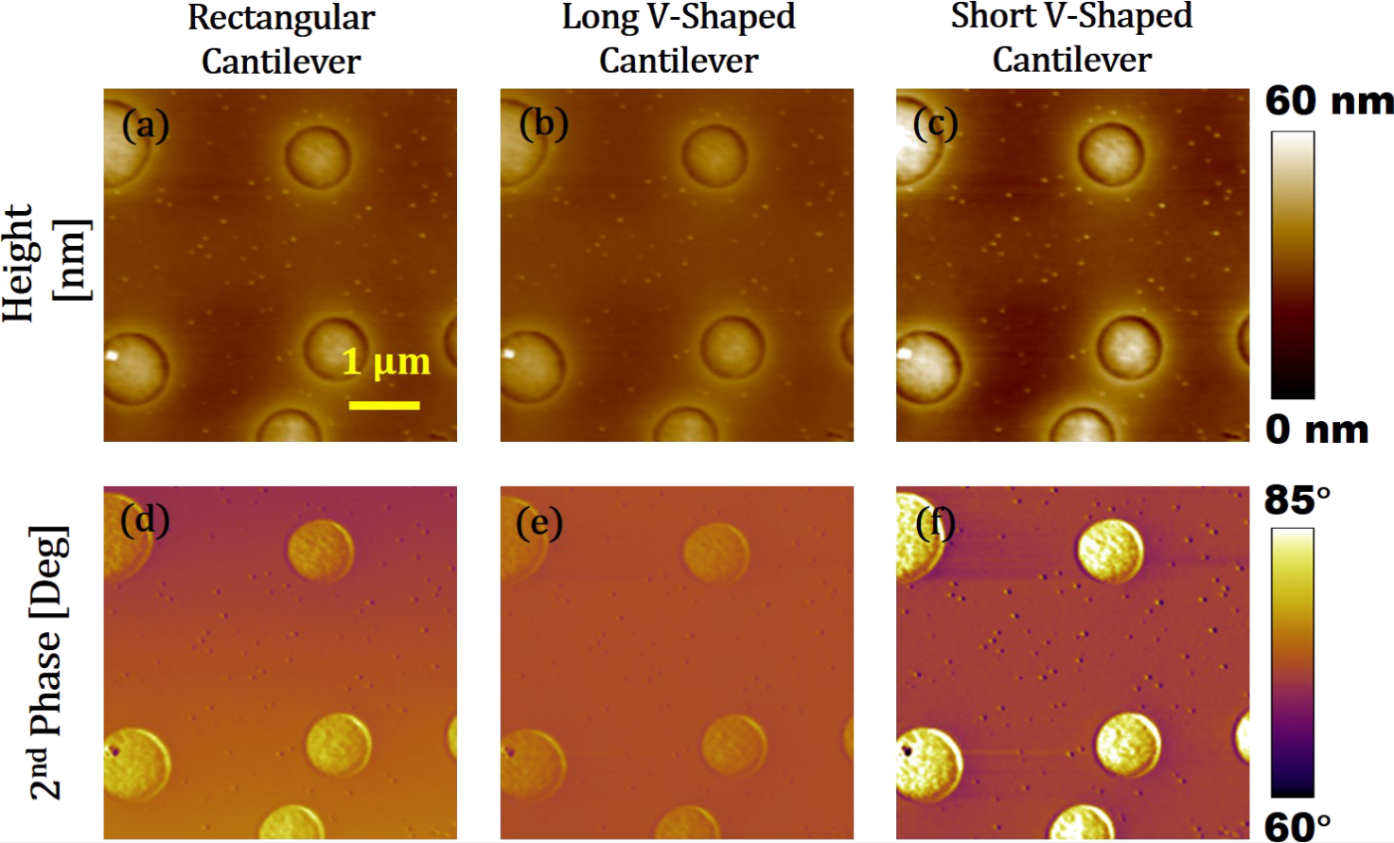
Bimodal AFM images of PS-LDPE samples. Left column: MULTI75G, middle column: TR400Long, right column: TR400Short. (a), (b), and (c) are height images. (d), (e), and (f) are phase images. All images were recorded at the same spot with a scan rate of 1 Hz, a setpoint ratio of 65%, a first eigenmode amplitude of *A*_1_ = 10 nm, and *A*_2_ = 2 nm. The gain was adjusted to maintain the quality of the images. The image size is 5 µm times 5 µm.

Figure 14a–c shows height or topography images of the PS–LDPE samples imaged using a rectangular, a long V-shaped, and a short V-shaped cantilever, respectively. There is a minor change in the topography. The circles of LDPE are brighter (higher topography) in Figure 14c than in Figure 14a and Figure 14b. The phase contrast for the second eigenmode is also provided in Figure 14d–f. The short V-shaped cantilever (Figure 14f) provides a higher phase contrast than the rectangular and the long V-shaped cantilever. The rectangular cantilever provides a higher contrast than the long V-shaped cantilever. The experimental results verify and complement the simulation results. It is important to mention that the simulation results considered both long-range attraction forces (i.e., van der Waals forces) and short-range repulsive forces (i.e., DMT model) while experimental work was done purely in the repulsive regime, in which material properties are more dominant on the dynamics of the cantilever. It should also be mentioned that the since all simulation studies were carried out at a setpoint around 60%, the tip–sample gap has been always higher that than the intermolecular distance causing the net forces to be attractive. Different tip–sample gaps can change the energies of the cantilever in different eigenmodes for bimodal AFM imaging. This can consequently affect the phase contrasts as discussed by Kiracofe and co-workers [47]. Hence, a higher phase contrast is observed in experiments compared to simulations.

It is important to note that the found optimum geometrical parameters of a V-shaped cantilever are correlated to each other. Therefore, there is a need for developing a normalized relationship with which the cantilever that can provide the maximum phase contrast can be determined. Based on the simulation results, an analytical relationship is developed as a model to provide general guidance in the selection of V-shaped cantilevers for bimodal AFM studies. It is shown in this study that the maximum phase contrast between PS and Au can be up to 19.4° in the 2nd eigenmode channel. However, this phase contrast is a theoretical value for the optimum dimensions. Commercially available cantilevers might have different dimension. Equation 22 is the relationship between the maximum phase contrast between Au and PS that can be observed given the dimensions:

[22] $$\Delta \phi_{\text{max}}=A\frac{b^{n}d^{p}}{L^{m}t^{q}}.$$

The constant values of *A*, *m*, *n*, *p* and *q* were constant values found by performing nonlinear regression and are shown in Table 7.

**Table 7:** Constants for Equation 22.

parameter	*A*	*m*	*n*	*p*	*q*

value	0.3573	0.3025	0.1833	0.8570	1.0471

For a selected V-shaped cantilever, one can have an estimate of the phase contrast observed in the second eigenmode. With the maximum theoretical phase contrast of 19.4°, the selected V-shaped cantilever can be compared to the best-case scenario (i.e., (Δϕ_max_/19.4)%) for any given sample.

## Conclusion

The effects of the geometrical dimensions of V-shaped cantilevers on static, dynamic, and vibrational parameters of higher bending modes and multifrequency atomic force microscope have been investigated. The parameters considered are cantilever length, based width, leg width and thickness. The ranges for each parameter are found by studying commercially available cantilevers. In order to find the optimum geometry, the cantilever is modeled as a Timoshenko beam and is excited simultaneously by two fundamental bending modes as bimodal AFM. In each step, the optimum dimensional parameter yielding the highest material composition contrast between two samples of gold (Au) and polystyrene (PS) is selected and used for the next round of simulation for optimizing the other geometrical parameters.

For cantilever lengths ranging from 85 to 310 µm, the ratio of frequency varies from 5.3 to 5.7 and the spring stiffness ratio changes from 13 to 15.5. For a rectangular cantilever these ratios are constant as 6.27 and 39.3, respectively, regardless of the geometrical parameters. Since the frequency ratio is smaller in comparison to rectangular cantilevers, the geometry of V-shaped cantilevers enhances the chance of self-excitation and makes these cantilevers more appropriate for multifrequency AFM, especially bimodal AFM. Furthermore, due to the lower spring constant of higher eigenmodes, lower forces are applied to surfaces. Hence, V-shaped cantilevers are better for imaging soft samples, such as biological cells or polymer samples, avoiding surface damage during imaging and characterization of material properties. When the length increases from 90 to 310 µm, the phase contrast decreases between two samples. The maximum phase contrast is observed for the V-shaped cantilever with a length of 90 µm.

Similarly, the cantilever base width is studied. As the base width increases from 74 to 254 µm, the phase contrast increases and the maximum phase contrast is observed when the base width is 254 µm. Having obtained the optimum length and base width, the leg width was varied in a range of 14 to 40 µm. For a leg width 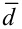 = 32 µm the maximum material contrast is achieved. The frequency ratio between the second and the first bending mode obtained for this value is 4.97. Since this ratio is close to the 5th harmonic, it can improve phase contrast in bimodal AFM. In comparison to another geometrical parameter, the leg width plays a crucial role in enhancing phase contrast, decreasing frequency ratio and spring constant ratio, which are important properties of the cantilever regarding tapping mode and multifrequency AFM. The last parameter that is optimized is the thickness. With the previously obtained optimum dimensions, *L*_opt_ = 90 µm, *b*_opt_ = 254 µm, and 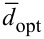 = 32 µm, a thickness range from 0.3 to 0.6 µm is analyzed. In comparison to the other dimensional parameters, thickness plays a minor role in changing frequency ratio, spring constant ratio and second mode phase contrast. The optimum thickness is 0.35 µm and the maximum material contrast for Au and PS is 19.4°. This is larger than the maximum contrast of an optimum rectangular cantilever. In previous studies, it was found that an optimum rectangular cantilever yields up to 7.57° of phase contrast between the same set of samples.

Having found the optimum theoretical V-shaped cantilever, three different commercial cantilevers are theoretically and experimentally studied. Two different types of commercially available V-shaped cantilevers are compared with a MULTI75G rectangular cantilever. The experiments were done on two different sets of samples (Au-PS and PS-LDPE). These experiments verified the trend found in simulations and provided more insight into the advantages and disadvantages for each cantilever.

Results show that after increasing the length of V-shaped cantilever from 100 to 200 µm, the phase contrast decreases significantly, which verifies our simulation results. The phase contrast images show that the maximum contrast is obtained with the short V-shaped cantilever. Although the short V-shaped cantilever is not very close in dimensions to the theoretically optimum dimensions according to our simulation results, it still provides a higher 2nd eigenmode phase contrast than the rectangular cantilever.

Finally, based on the simulation results, a nonlinear equation is derived by regression as analytical model that can help AFM users in selecting V-shaped cantilevers in bimodal AFM. Given the dimensions of the V-shaped cantilevers, the achievable percentage of the optimized phase contrast can be calculated. Selecting the right V-shaped cantilever involves considering different geometrical parameters that can directly influence the quality of bimodal AFM measurements. This nonlinear equation provides a single value to quantify how suitable the cantilever is for a given measurement.

## Supporting Information

File 1 Simulated tip–sample force interactions.
