# Supplementary material for: Design of V-shaped cantilevers for enhanced multifrequency AFM measurements
Source: Beilstein J Nanotechnol. 2020 Oct 6;11:1525–41. doi: 10.3762/bjnano.11.135 (PMC7554663; doi:10.3762/bjnano.11.135)
Supplement: File 1 — Simulated tip–sample force interactions. [file Beilstein_J_Nanotechnol-11-1525-s001.pdf]

BEILSTEIN JOURNAL OF NANOTECHNOLOGY

Supporting Information

for

Design of V-shaped cantilevers for enhanced multifrequency AFM measurements

Mehrnoosh Damircheli and Babak Eslami

Beilstein J. Nanotechnol. **2020,** *11,* 1525–1541. doi:10.3762/bjnano.11.135

Simulated tip–sample force interactions

License and Terms: This is a supporting information file under the terms of the Creative Commons Attribution License (https://creativecommons.org/licenses/by/4.0). Please note that the reuse, redistribution and reproduction in particular requires that the authors and source are credited.
The license is subject to the *Beilstein Journal of Nanotechnology* terms and conditions: (https://www.beilstein-journals.org/bjnano)

Figure S1 presents more detailed information about the tip–sample interactions. As provided in the main manuscript, the tip–sample forces are modeled as long-range van der Waals forces and short-range DMT repulsion. Figure S1c shows that the net tip–sample forces on the Au surface gets to repulsive regime (phase values smaller than 90°) for low setpoint values. Otherwise, the net total force interactions remain in the attractive regime since, based on the given setpoint, the distance value is higher that the intermolecular value of 0.165 nm. It should be mentioned that this simulation model does not include experimental factors such as adhesion forces due to humidity or electrostatic charges. Therefore, there can be other factors that can change the overall behavior of the force interactions in actual experiments but this model can provide a reliable general guideline for understanding the dynamics of the cantilever while interacting with Au and PS surfaces in bimodal AFM with a V-shaped cantilever.

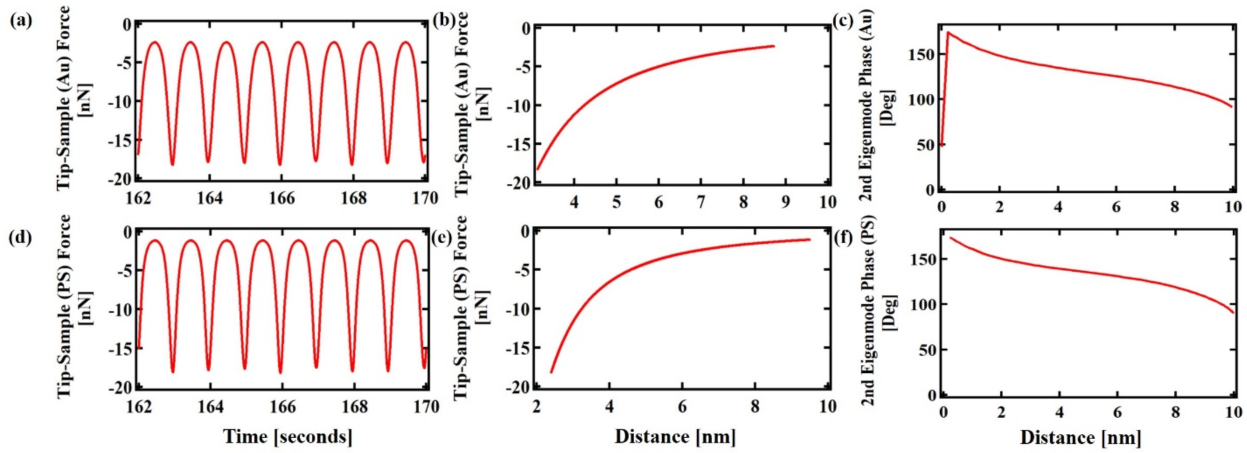

Figure S1: Simulation results for bimodal AFM for V-shaped cantilever with $L = 90$ μm, $\bar{d}_{\text{ref}} = 15$ μm, $b_{\text{ref}} = 86$ μm, $t_{\text{ref}} = 0.4$ μm: (a), (b) and (c) are the tip–sample force versus time, tip sample force versus distance, and 2nd eigenmode phase versus distance curves for Au respectively. (d), (e) and (f) are the tip–sample force versus time, tip–sample force versus distance, and 2nd eigenmode phase versus distance curves for PS.
